# ENDS Flavor Preference by Menthol Cigarette Smoking Status among US Adults, 2018–2019

**DOI:** 10.3390/ijerph18010240

**Published:** 2020-12-31

**Authors:** Brian L. Rostron, Joanne T. Chang, Cindy M. Chang, Rebecca A. Jackson, Bridget K. Ambrose

**Affiliations:** Center for Tobacco Products, US Food and Drug Administration, Silver Spring, MD 20993, USA; joanne.chang@fda.hhs.gov (J.T.C.); cindy.chang@fda.hhs.gov (C.M.C.); rebecca.jackson@fda.hhs.gov (R.A.J.); bridget.ambrose@fda.hhs.gov (B.K.A.)

**Keywords:** e-cigarette, flavor, menthol, tobacco

## Abstract

E-cigarette flavor preference may differ among smokers using e-cigarettes, but little information is available on preferences by menthol cigarette status. Using nationally representative data for US adults from the 2018–2019 Tobacco Use Supplement to the Current Population Survey, we analyzed e-cigarette flavor preference by menthol cigarette status and e-cigarette device type for dual-cigarette and e-cigarette users and e-cigarette users who had recently quit smoking by trying to switch to e-cigarettes (“switchers”). Approximately half (52.2%) of dual users of menthol cigarettes and e-cigarettes reported using menthol/mint-flavored e-cigarettes as did 41.4% of “switchers” who had smoked menthol cigarettes; exclusive menthol/mint flavor use was 13.1% for dual users and 21.3% for “switchers.” A similar proportion (45.1%) of dual users who smoked nonmenthol cigarettes used tobacco-flavored e-cigarettes, but only 26.7% of “switchers” who had smoked nonmenthol cigarettes used tobacco-flavored e-cigarettes. Approximately 60% of dual users and “switchers” used fruit/other flavors, regardless of menthol cigarette use. By device type, 63.9% of dual users of cartridge-based e-cigarettes and menthol cigarettes used menthol/mint-flavored e-cigarettes. Approximately 75% of dual users and 85% of “switchers” who used tank or mod systems used fruit/other flavors. Menthol cigarette smokers may be particularly likely to use menthol/mint e-cigarettes, and a majority of dual users and “switchers” used fruit/other flavors. These results can inform policy measures concerning flavored electronic nicotine delivery system products.

## 1. Introduction

Flavor use and preference among electronic nicotine delivery system (ENDS) users is an important public health issue with implications for tobacco use and harm [1,2,3,4]. Certain flavors such as tobacco and menthol may differentially appeal to adult cigarette smokers trying to transition from combustible tobacco use, whereas young ENDS users may tend to prefer sweeter flavors such as candy or fruit [5,6,7,8,9,10,11]. In January 2020, the US Food and Drug Administration (FDA) issued a final guidance document that stated that the agency would prioritize regulatory enforcement against cartridge-based ENDS with flavors other than tobacco or menthol [12]. At the time, the agency noted that adolescent ENDS users were more likely to use flavors such as fruit and mint rather than tobacco and menthol. This guidance sought to protect population health by striking a balance between maintaining e-cigarettes as an alternative for adult combusted tobacco users while dissuading youth use.

These conclusions are consistent with data from nationally representative surveys. In Wave 2 of the Population Assessment of Tobacco and Health (PATH) Study from 2014 to 2015, tobacco flavor was used by 50.5% of adult ENDS users aged 25 years and older who used a single flavor, menthol/mint was used by 23.3%, fruit by 15.9%, and candy or sweets by 7.8%. In contrast, among youth aged 12 to 17 years, the comparable figures were 43.3% for fruit, 31.2% for tobacco, 16.5% for candy or sweets, and 4.8% for menthol/mint. Multivariable logistic regression analysis of Wave 2 data found that youth and young adults were greater than three times more likely to use fruit- and candy-flavored e-cigarettes than older adults [9]. Additional analysis of Wave 2 data found that menthol/mint was the most commonly used flavor among adult ENDS users who reported using a single nontobacco flavor at 37.4% but was only used by 6.1% of youth who used a single nontobacco flavor [13]. Overall, 40.7% of adults aged 25 years and older who had used a flavored e-cigarette in the past 30 days had used menthol/mint flavor compared to 20.4% of youth users aged 12 to 17 years [5].

More specifically, it is possible that mint flavor has had greater appeal among youth than menthol. In 2019 Monitoring the Future data, the proportions of 8th, 10th, and 12th grade JUUL users who reported that the flavor that they usually used with the device was mint were 29.2%, 43.5%, and 47.1% [14]. The comparable figures for menthol were less than 6.0% for each grade. In 2020 National Youth Tobacco Survey data, the flavors most commonly used by high school students who used flavored e-cigarettes were fruit at 73.1%, mint at 55.8%, menthol at 37.0%, and candy, desserts, or other sweets at 36.4%. Among middle school students, the most commonly used flavors were fruit at 75.6%, candy, desserts, or other sweets at 47.2%, mint at 46.5%, and menthol at 23.5% [15]. 

Although the existing literature suggests a differential appeal of flavored e-cigarette use by age, it is less clear whether adult menthol cigarette smokers are more likely to use menthol-flavored ENDS than nonmenthol smokers, and if so whether such a preference is consistent across ENDS device types. 

In this study, we analyze e-cigarette flavor preference by menthol cigarette smoking status among US adults using data from the 2018–2019 Tobacco Use Supplement to the Current Population Survey (TUS-CPS). We examine this flavor preference among dual-cigarette and e-cigarette users and recent former cigarette smokers who report switching to e-cigarettes to try to quit smoking. We also analyze e-cigarette flavor preference by menthol smoking by device type for disposable, cartridge-based, and tank or modifiable products.

## 2. Materials and Methods 

The TUS-CPS is a nationally representative survey of tobacco use behaviors and attitudes among US adults aged 18 years and older sponsored by the National Cancer Institute and also funded by the Food and Drug Administration [16]. The most recent survey wave was conducted in July 2018, January 2019, and May 2019. All information in this analysis was self-reported by survey participants. We analyzed e-cigarette flavor preference among (1) current dual-cigarette and e-cigarette users and (2) current e-cigarette users who quit cigarette smoking completely in the past 12 months and reported quitting smoking while trying to quit by switching to e-cigarettes, even if they did not think that it was effective (“switchers”). Current smokers and “switchers” reported having smoked at least 100 cigarettes in their lives, with current smokers reporting that they currently smoked every day or some days and “switchers” reporting that they had smoked 12 months ago and currently no longer smoked at all. Current e-cigarette users reported using e-cigarettes every day or some days. We further divided these groups by menthol or nonmenthol cigarette preference as a smoker. Results are not presented for the 3.8% of dual users and 3.9% “switchers” who reported that they did not have a usual cigarette type. A small number (*n* = 31) of current e-cigarette users who had quit smoking in the past 12 months did not specifically report that they had used e-cigarettes to quit smoking, but the addition of these participants to the “switcher” group did not substantively affect estimates.

E-cigarette users were asked whether their e-cigarette was usually flavored and, if so, whether the flavor was usually tobacco; mint or menthol; clove, spice, herb, fruit, alcohol, candy, sweets, or chocolate; or some other flavor. Respondents could select one or more of these flavor options. Participants who said their e-cigarette was not flavored were specifically asked whether their e-cigarette was usually tobacco flavored. E-cigarette flavor preference was categorized in this analysis as any and exclusive menthol/mint use; any and exclusive tobacco flavor use; any and exclusive fruit or other flavor use; and no flavor use. These categories are not mutually exclusive, and estimates do not sum to 100%. We conducted a similar analysis stratified by user-reported device type in which the device categories were disposable e-cigarette, e-cigarette with replaceable prefilled cartridges, and e-cigarette with a refillable tank or a modifiable or mod system with components such as batteries or atomizers that the user can customize. Devices in this last category are often referred to as open system devices in the research literature [17]. Associations between ENDS flavor use and menthol cigarette smoking were analyzed using chi-squared tests of the proportions of menthol and nonmenthol cigarette smokers who used various flavors with e-cigarettes.

The TUS-CPS data were analyzed using replicate weights to account for sampling probabilities and the complex survey design. Balanced repeated replication was used with a Fay’s adjustment value of 0.5. Estimates with a relative standard error of at least 30% were flagged for possible imprecision, and results for the small number of “switchers” who reported using disposable e-cigarettes (*n* = 7 for both those who had used menthol and nonmenthol cigarettes) are not presented due to small sample size. 

## 3. Results

Table 1 presents e-cigarette flavor preference by menthol cigarette smoking status. One half (52.2%) of dual users of menthol cigarettes and e-cigarettes reported using menthol/mint-flavored e-cigarettes as did 41.4% of “switchers” who had smoked menthol cigarettes. Exclusive menthol/mint flavor use was 13.1% among dual users and 21.3% among “switchers.” Menthol cigarette smoking dual users were consistently more likely to use menthol/mint-flavored e-cigarettes and less likely to use or exclusively use tobacco-flavored e-cigarettes than nonmenthol smoking dual users. Menthol/mint flavor use was much less common among those who had smoked nonmenthol cigarettes, with 10.3% for dual users and 21.5% for “switchers.” Among dual users who used nonmenthol cigarettes, 32.3% and 45.1% reported exclusive and any tobacco-flavored e-cigarette use compared to 17.9% and 26.7% among “switchers” who had smoked nonmenthol cigarettes. 

Approximately 60% of dual users and “switchers” used fruit or other e-cigarette flavors, regardless of menthol cigarette preference. Exclusive use of these flavors was highest among “switchers” who had used nonmenthol cigarettes at 52.0% and lowest among dual users who used menthol cigarettes at 34.5%.

Table 2 shows e-cigarette flavor preference by device type and menthol smoking status. Dual users who smoked menthol cigarettes were more likely to use menthol/mint-flavored e-cigarettes than nonmenthol smoking dual users across e-cigarette device types. Menthol/mint flavor use was more common among users of e-cigarettes with cartridges at 63.9% and less common among open system (i.e., tank or mod system) users at 44.3% (*p* = 0.005). Exclusive menthol/mint flavor use varied from 8.2% among disposable e-cigarette users and 9.2% among tank or mod users to 20.6% among users of cartridge-based devices. Approximately half of “switchers” to cartridge-based e-cigarettes who had used menthol cigarettes used menthol/mint-flavored ENDS (50.5%), and the proportion for open system users was 37.5%. The corresponding estimates for exclusive menthol/mint use were 35.6% for cartridge-based device users and 11.5% for open system users.

A large proportion of “switchers” who used open system devices used fruit or other flavors. Any use of these flavors was 88.5% for former nonmenthol smokers and 82.9% for former menthol smokers, and exclusive use was 72.7% and 59.1%, respectively. Among dual users who used open system devices, any use of these flavors was 73.4% and 75.7% for menthol and nonmenthol smokers, and exclusive use was 47.9% and 61.6%, respectively. Use of fruit or other flavors was less common among cartridge-based e-cigarette users with about 40% of these dual users and “switchers” using them, regardless of menthol cigarette use, although sample size was limited for “switchers.”

## 4. Discussion

In a nationally representative survey of the US adult population, a large proportion of e-cigarette users who had used or were using menthol cigarettes reported using menthol/mint-flavored e-cigarettes. Exclusive use of these flavors in this group was more limited, although it was most common among users of cartridge-based devices. Exclusive tobacco-flavored e-cigarette use was fairly common among dual users who smoked nonmenthol cigarettes. Any and exclusive use of fruit and other flavors was common among dual users and “switchers,” with use of these flavors particularly prevalent among users of open system tank or mod devices.

These TUS-CPS data were collected in 2018 and 2019, approximately seven to seventeen months before the release of the FDA final guidance document on flavored ENDS [12]. As such, the estimates represent a cross-sectional “snapshot” of ENDS flavor use among US adults before introduction of this enforcement policy. The estimates are intended to provide understanding of product use patterns within the context of a rapidly evolving tobacco marketplace and regulatory environment.

The availability of flavors may encourage ENDS initiation and use, particularly among young people. Kowitt et al. and Huang et al. conducted systematic reviews of studies of perceptions and use of nonmenthol-flavored tobacco products including ENDS and found that adolescents and young adults had positive perceptions of these flavored products [18,19]. Study participants also cited flavors as a reason for tobacco product experimentation and use. Meernik et al. subsequently produced an updated review that looked specifically at nonmenthol-flavored ENDS and found that flavors increased product appeal and willingness to try and use ENDS and decreased harm perceptions of ENDS use among youth [20]. They also found among adults that flavors increased product appeal and were cited as a reason for ENDS use but that the effects of flavors on smoking cessation were not clear.

The findings in this study are consistent with more limited results from previous studies, although our analysis has the strength of using data from a nationally representative and larger study population. McQueen, Tower, and Sumner interviewed 15 “vaping enthusiasts” who generally reported that new e-cigarette users often began with devices that looked and felt like cigarettes and also often initiated use with tobacco or menthol flavor [21]. Webb, Hooper, and Smiley surveyed 148 adult current cigarette smokers in South Florida who were ever e-cigarette users and found in multivariable analysis that menthol smokers were more than six and a half times more likely to have used menthol-flavored e-cigarettes in the past 30 days than nonmenthol smokers [22]. 

Use of ENDS and various flavors and device types has changed dramatically in the US in recent years [1,15,23]. Much of the increase in youth use of ENDS has occurred with cartridge-based products such as JUUL [24,25]. As noted, the US FDA announced in January 2020 that it would be exercising regulatory enforcement against these products with flavors other than tobacco or menthol [12]. Several states and localities have enacted various policies limiting the sale of flavored tobacco products including ENDS [26]. It remains to be seen what effect these measures will have on ENDS use overall and by flavor and device type, particularly among adult smokers who may be using ENDS to transition from combusted tobacco use.

The results in this study can inform policy measures concerning flavored ENDS products generally. They provide evidence that among adults menthol cigarette smokers are particularly likely to use menthol or mint-flavored e-cigarettes compared to nonmenthol smokers. This association may inform efforts to identify product characteristics that encourage adult smokers to transition from combusted tobacco use. 

One limitation of this study is that all product use and characteristics information was self‑reported by survey participants and may include some misreporting. For example, a relatively small proportion of e-cigarette users reported using no flavor. It is not entirely clear to what extent such products were truly unflavored or if participants did not know that they were flavored. ENDS users were asked to report their usual flavor and could select multiple options, but some participants may have not reported every flavor that they used. Sample size was also limited for some device type categories, especially flavor preference for “switchers” who used disposable e-cigarettes and to some extent cartridge-based devices. “Menthol/mint” was a single response option for e-cigarette flavor in the TUS-CPS survey, so we cannot differentiate between use of these two flavors. Future research can assess the effect of enforcement policies and other measures and developments on flavor preferences among adult cigarette and ENDS users. Additional information about device type users such as “switchers” who use disposable and cartridge-based e-cigarettes could be useful. Lastly, the cross-sectional nature of TUS-CPS data limits our ability to perform such an analysis, but prospective longitudinal studies such as PATH can assess whether use of specific ENDS flavors is associated with complete switching or dual use among menthol and nonmenthol cigarette smokers.

## 5. Conclusions

Menthol cigarette smokers are particularly likely to use menthol/mint-flavored e-cigarettes compared to nonmenthol smokers. A majority of dual users and “switchers” used fruit/other flavors regardless of menthol cigarette use. These results can inform policy measures concerning flavored ENDS products. 

## Disclaimer

The findings and conclusions in this report are those of the authors and do not necessarily represent the official position of the Food and Drug Administration.

## Figures and Tables

**Table 1:** E-Cigarette Flavor Use by Menthol Cigarette Smoking Status among US Adults (18+), 2018–2019 (% and 95% Confidence Interval).

ENDS Flavor Type	Dual Users of Cigarettes and E-Cigarettes		E-Cigarette Users Who Quit Smoking While Switching to E-Cigarettes (“Switchers”)	
	Use Menthol Cigarettes (*n* = 352)	Use Nonmenthol Cigarettes (*n* = 752)	Used Menthol Cigarettes (*n* = 99)	Used Nonmenthol Cigarettes (*n* = 208)
Any menthol/mint flavor use	52.2 (45.3, 59.0) ^	10.3 (7.2, 14.4)	41.4 (30.3, 53.5) ^	21.5 (15.1, 29.8)
Exclusive menthol/mint flavor use	13.1 (9.7, 17.4) ^	2.7 (1.4, 5.0) *	21.3 (12.5, 33.8)	8.9 (4.8, 15.8) *
Any other menthol/mint flavor use	39.1 (32.7, 46.0) ^	7.6 (4.9, 11.8)	20.1 (12.9, 30.0)	12.7 (7.5, 20.6)
Any tobacco flavor use	28.9 (23.2, 35.4) ^	45.1 (40.6, 49.6)	18.4 (11.5, 28.3)	26.7 (20.3, 34.4)
Exclusive tobacco flavor use	6.9 (4.5, 10.4) ^	32.3 (28.5, 36.4)	7.5 (3.6, 15.1) *^	17.9 (12.8, 24.5)
Any other tobacco flavor use	22.0 (16.9, 28.3) ^	12.7 (9.4, 17.1)	10.9 (6.0, 18.9)	8.9 (5.2, 14.7)
Any fruit or other flavor ^1^ use	59.8 (53.3, 65.9)	57.1 (52.2, 61.8)	64.1 (52.7, 74.2)	66.9 (58.4, 74.3)
Exclusive fruit or other flavor use	34.5 (28.4, 41.2)	43.7 (38.7, 48.9)	44.5 (33.5, 56.1)	52.0 (42.8, 61.0)
Any other fruit or other flavor use	25.3 (20.1, 31.2) ^	13.4 (10.3, 17.3)	19.6 (12.4, 29.6)	14.9 (9.3, 23.0)
No flavor	5.2 (2.8, 9.4) *	5.3 (3.8, 7.3) *	3.2 (1.0, 9.5) *	4.2 (2.3, 7.7) *

^1^ Fruit or other flavor includes clove, spice, herb, fruit, alcohol, candy, sweets, chocolate, or “some other type”. * Relative standard error ≥ 30%. ^ *p* < 0.05 for difference of proportions for menthol and nonmenthol cigarette users. Flavor use overall shown in gray.

**Table 2:** E-Cigarette Flavor Use among Dual Users and “Switchers” by Device Type and Menthol Cigarette Smoking Status among US Adults (18+), 2018–2019 (% and 95% Confidence Interval).

ENDS Flavor Type	Dual Users of Cigarettes and Disposable E-Cigarettes		Dual Users of Cigarettes and Cartridge-Based E-Cigarettes		Dual Users of Cigarettes and Tank or Mod E-Cigarettes	
	Use Menthol Cigarettes (*n* = 34) *	Use Nonmenthol Cigarettes (*n* = 77)	Use Menthol Cigarettes (*n* = 114)	Use Nonmenthol Cigarettes (*n* = 273)	Use Menthol Cigarettes (*n* = 184)	Use Nonmenthol Cigarettes (*n* = 372)
Any menthol/mint flavor use	51.8 (31.0, 72.0) *^	7.6 (2.9, 18.7) *	63.9 (52.7, 73.7) ^	14.6 (9.4, 21.9)	44.3 (35.2, 53.8) ^	6.1 (3.1, 11.7)
Exclusive menthol/mint flavor use	8.2 (3.3, 18.9) *	1.3 (0.2, 7.8) *	20.6 (13.8, 29.5) ^	4.1 (2.0, 8.2) *	9.2 (5.3, 15.3) ^	0.6 (0.1, 2.5) *
Any other menthol/mint use	43.6 (24.3, 65.0) *^	6.3 (2.0, 17.8) *	43.3 (32.5, 54.8) ^	10.5 (5.5, 18.9) *	35.2 (26.8, 44.6) ^	5.5 (2.6, 11.2) *
Any tobacco flavor use	49.3 (28.9, 69.9) *	72.2 (58.3, 82.8)	39.5 (29.8, 50.1) ^	53.9 (46.4, 61.3)	17.2 (11.3, 25.3) ^	32.7 (27.1, 38.9)
Exclusive tobacco flavor use	12.1 (4.6, 28.5) *^	54.6 (41.2, 67.4)	14.2 (8.9, 22.0) ^	39.7 (33.1, 46.8)	1.2 (0.3, 5.5) *^	22.7 (18.0, 28.2)
Any other tobacco flavor use	37.2 (19.4, 59.4) *	17.5 (9.3, 30.6) *	25.2 (49.9, 70.2)	14.2 (8.4, 22.9)	16.0 (10.3, 23.9)	10.1 (6.3, 15.6)
Any fruit or other flavor ^1^ use	51.1 (32.1, 69.9) *	34.8 (22.8, 49.0)	40.2 (29.1, 52.4)	43.6 (36.2, 51.4)	75.7 (66.4, 83.1)	73.4 (67.8, 78.3)
Exclusive fruit or other flavor use	31.7 (13.8, 57.2) *	20.9 (11.3, 35.0)	16.8 (9.0, 29.2) *	28.8 (22.0, 36.7)	47.9 (38.9, 57.1) ^	61.6 (55.3, 67.6)
Any other fruit or other flavor use	19.5 (7.1, 43.2) *	13.9 (6.6, 26.7) *	23.4 (15.6, 33.6)	14.8 (9.8, 21.8)	27.8 (20.3, 36.7) ^	11.8 (7.8, 17.5)
No flavor	4.4 (1.1, 16.1) *	5.6 (2.4, 12.8) *	5.0 (1.5, 15.2) *	7.6 (4.9, 11.7)	4.1 (1.4, 11.1) *	2.9 (1.6, 5.1)
**ENDS Flavor Type**	**“Switchers” ^2^ Who Use Disposable** **E-Cigarettes ^3^**		**“Switchers” Who Use Cartridge-Based** **E-Cigarettes**		**“Switchers” Who Use Tank or Mod** **E-Cigarettes**	
	**Used Menthol Cigarettes (*n* = 7)**	**Used Nonmenthol Cigarettes (*n* = 7)**	Used Menthol Cigarettes (*n* = 34) *	**Used Nonmenthol Cigarettes (*n* = 80)**	**Used Menthol Cigarettes (*n* = 54)**	**Use Nonmenthol Cigarettes (*n* = 118)**
Any menthol/mint flavor use	-	-	50.5 (31.9, 68.9) *	33.9 (22.8, 47.0)	37.5 (23.0, 54.6) ^	14.6 (7.3, 27.0) *
Exclusive menthol/mint flavor use	-	-	35.6 (18.1, 58.0) *	20.0 (10.9, 33.7)	11.5 (4.7, 25.7) *	1.7 (0.3, 9.3) *
Any other menthol/mint use	-	-	14.9 (5.7, 33.5) *	13.9 (7.0, 25.7) *	25.9 (14.8, 41.3)	12.8 (6.1, 25.0) *
Any tobacco flavor use	-	-	30.8 (15.2, 52.6) *	42.8 (31.0, 55.5)	7.9 (3.3, 17.8)	13.1 (7.5, 22.0)
Exclusive tobacco flavor use	-	-	13.5 (5.1, 31.2) *	30.8 (20.7, 43.2)	1.0 (0.1, 7.0) *^	7.2 (3.4, 14.8) *
Any other tobacco flavor use	-	-	17.4 (7.0, 36.9) *	12.1 (5.8, 23.5) *	7.0 (2.7, 16.8) *	5.9 (2.7, 12.3) *
Any fruit or other flavor use	-	-	40.1 (23.6, 59.1) *	38.4 (26.2, 52.2)	82.9 (67.5, 91.8)	88.5 (79.8, 93.7)
Exclusive fruit or other flavor use	-	-	24.5 (10.7, 46.7) *	25.0 (14.8, 39.0)	59.1 (42.1, 74.1)	72.7 (59.5, 82.9)
Any other fruit or other flavor use	-	-	15.6 (6.3, 33.8) *	13.4 (6.5, 25.7) *	23.8 (13.1, 39.4)	15.7 (8.3, 28.0) *
No flavor	-	-	2.8 (0.5, 15.2) *	6.6 (3.2, 13.2) *	2.5 (0.4, 15.5) *	1.6 (0.4, 7.1) *

^1^ Fruit or other flavor includes clove, spice, herb, fruit, alcohol, candy, sweets, chocolate, or “some other type”. ^2^ “Switchers” reported that they quit cigarette smoking completely in the past 12 months and tried to quit by switching to e-cigarettes. ^3^ Results are not presented for “Switchers” who use disposable e-cigarettes due to small sample size. * Relative standard error ≥ 30% or unweighted denominator < 50. ^ *p* < 0.05 for difference of proportions for menthol and nonmenthol cigarette users. Flavor use overall shown in gray.

## Data Availability

Data are publicly available at https://cancercontrol.cancer.gov/brp/tcrb/tus-cps.
